# Animal Models in Alcohol Research

**Published:** 2000

**Authors:** Boris Tabakoff, Paula L. Hoffman

**Affiliations:** Boris Tabakoff, Ph.D., is a professor and chair and Paula L. Hoffman, Ph.D., is a professor in the Department of Pharmacology, University of Colorado Health Sciences Center, Denver, Colorado

**Keywords:** animal model, scientific model, research, AOD (alcohol or other drug) dependence, AOD tolerance, body part, animal selectively bred for AOD preference, quantitative trait locus, route of administration, theory of AODU (AOD use, abuse, and dependence)

## Abstract

Animal models are important tools in the study of alcohol use, abuse, and dependence because they allow researchers to use methods that cannot be used with human subjects. Animal models have been developed to study various aspects of alcohol use and dependence, including alcohol-seeking behavior, alcohol-related organ damage, tolerance to alcohol, and physical dependence on alcohol. Because animal models can be genetically manipulated, they are also valuable for research into the genetic determinants of alcoholism. Issues surrounding the use of animal models in alcohol research include the species of animal used, the method of alcohol administration, and the model’s face and predictive validity.

Because of ethical concerns and experimental difficulties in studying alcoholism in humans, a substantial portion of research on the topic of alcohol intoxication and dependence has used nonhuman animals as experimental models. A model, in this sense, refers to something that is used to help visualize that which cannot be directly observed. In other words, by using experimental animals, scientists are attempting to dissect the complex disorder of alcoholism, in part by breaking it down into its component behaviors and studying the determinants of those behaviors.

The behaviors that characterize alcoholism in humans, according to the criteria for diagnosing alcoholism outlined in the *Diagnostic and Statistical Manual of Mental Disorders, Fourth Edition (DSM–IV)* (American Psychiatric Association 1994), include the following: (1) tolerance, or the need for increased amounts of alcohol to obtain the desired effects; (2) withdrawal symptoms after discontinuation of alcohol use; (3) taking alcohol in large amounts over periods longer than initially intended; (4) persistent desire or unsuccessful efforts to decrease alcohol use; (5) spending a great deal of time acquiring alcohol; (6) reducing important social and occupational activities because of alcohol use; and (7) continued use despite a recurrent physical or psychological problem associated with alcohol use.

One system (A) is a model for another system (B) if the study of A furthers the understanding of B, regardless of any causal connection between them (Kaplan 1964; McClearn 1988). For the model to be efficient, system A should be simpler than system B (McClearn 1988). Therefore, the animal models that are most commonly used in alcohol research have been designed in an attempt to understand, at the physiological, biochemical, or molecular level, the basis for a particular behavior that is believed to be an analog of a behavior associated with human alcoholism.

This article discusses the advantages of using animal models, especially in alcohol research, presents issues related to the development and use of animal models of alcoholism, and describes various animal models that have been developed to study aspects of alcoholism. We have focused, for the most part, on animal models of excessive alcohol intake, because this is the key factor that leads to organ damage and alcohol dependence. The crucial question to be answered using these models is the following: Why do some people consume alcohol in quantities that are injurious to themselves and to those around them? We also describe in a more limited way certain models that are used to determine how alcohol produces damage to various organs. These descriptions are included as examples of models that can be used to understand the *results* of excessive alcohol drinking, rather than the mechanisms which motivate people to drink alcohol.

## Advantages of Animal Models

Animal models allow researchers to use methods that would be unethical with human subjects. In some cases, using humans in alcohol research (and in research on other drugs of abuse) raises specific ethical issues, such as the risk involved in administering an addictive drug to humans and the related risks of accidents and of medical and psychological consequences. Clearly, these risks can be circumvented in animal studies. Because of the risks inherent in human alcohol studies, as well as the limitations imposed when human subjects are used, animal model studies have been, and continue to be, invaluable for addressing the basic questions of alcohol research.

There are also scientific reasons to use animal models. Although cells and tissues can be used for biochemical and molecular biological studies, there is no way to relate the results of these studies directly to any particular behavior. Therefore, research into determinants of behavior can best be carried out in experimental animals. A hallmark and an advantage of animal research, especially research into complex disorders such as alcoholism, is that it has the effect of simplifying complex behaviors by producing models that are relevant to the human situation.

Both human and animal studies indicate that genetic factors play a role in the development of alcoholism, leading researchers to focus on identifying genes associated with alcoholism or a predisposition to alcoholism. Research in this area has benefited from the use of genetic engineering techniques.

## Validity of Animal Models

By using animal models to study human disorders, we are implicitly acknowledging the evolutionary relationship between humans and other animals. There is abundant evidence that various vertebrate (and even invertebrate) species have similar biochemical and physiological systems, although sometimes these systems have different purposes. In addition, embryonic development in animals is similar to that of humans, allowing the study of the effects of alcohol and other drugs on this development. Nevertheless, animal species differ, and the relevance of results obtained using animal models to the human situation may depend on the species chosen. In addition, animal models may have either face validity (i.e., they mimic some aspect of the human condition) or predictive validity (i.e., results obtained with the animal model are predictive of alcohol’s actions or of treatment efficacy in humans). We would expect that animals most closely related to humans genetically or evolutionarily (e.g., non-human primates), would provide the best face validity. However, studies of alcohol effects in nonhuman primates are very expensive and technically difficult. Fortunately, many rodent models have been developed to study both the causes of human diseases and the efficacy of treatments (e.g., mouse models for anticonvulsant drugs, rat models for antidepressant drugs). In these cases, the models have predictive validity (i.e., medications that are effective in the model are also effective in humans).

## Animal Models of Alcoholism: Dependence, Organ Damage, and Behavioral Consequences

Animal models of many aspects of human alcoholism have been developed. In these models, which primarily use rodents such as mice and rats, the researcher usually controls alcohol intake. Alcohol may be fed to the animals in a liquid diet as their sole source of nutrition, administered by a tube implanted into the stomach (i.e., intragastric administration) or by injection, or administered through inhalation in specially designed chambers. The goal of these types of models is to generate the adaptive changes in the brain that are associated with the development of tolerance to and physical dependence on alcohol, both of which can be assessed in the animals.

### Dependence and Tolerance

The characteristics of alcohol dependence and tolerance in mice and rats are similar to those exhibited by humans. By measuring tolerance- or dependence-related changes in brain biochemistry and gene expression in animal models, researchers have made progress in identifying the neurochemical systems associated with alcohol tolerance and physical dependence, and have introduced a novel concept indicating that alcohol withdrawal can produce brain damage. However, most of these models lack a key element of the human situation because the researcher, rather than the animal, determines the alcohol intake. In addition, environmental elements play a role in tolerance. A person may exhibit tolerance to the effects of alcohol while drinking in a favorite restaurant or bar, but at the same blood alcohol concentration, may not exhibit tolerance when in a completely novel environment, such as when driving on an unfamiliar highway. Although a number of studies have tried to model environmental influences on the expression of alcohol tolerance or physical dependence in animals, the measure of environmental influence is not a major component of many studies of tolerance, dependence, or other consequences of alcohol ingestion.

### Organ Damage

Animal models have also been used to examine the mechanisms by which alcohol produces organ damage (Lieber et al. 1989). Both rat and baboon models have proven useful for studying the toxic effects of alcohol on the liver and how these effects vary with nutrition. Lieber and colleagues (1965) fed alcohol to rats as part of a nutritionally adequate liquid diet containing relatively large amounts of fat, but no liver lesions more advanced than fatty liver could be produced by this method. Tsukamoto and colleagues (1986) and French and colleagues (1986) produced more severe liver damage in rats with continuous intragastric administration of alcohol and a nutritionally defined low-fat liquid diet, and this damage was increased by increasing the fat content of the diet. This method produced a model of alcoholic liver disease in rats that is more comparable to the disease that occurs in humans. Lieber and DeCarli (1974) also used a liquid diet feeding technique with baboons. In this model, the biochemical and morphological changes in the liver, including cirrhosis, mimic those seen in human alcoholics, although alcoholic hepatitis is not observed. Discrepancies in the ability to produce alcoholic liver disease using this model (e.g., Rogers et al. 1981; Mezey et al. 1980, 1983) may be due to the nutrient value of the diets used or to the fact that some studies used a small number of animals (Lieber and DeCarli 1991). These findings illustrate the importance of the choice of species for animal studies using alcohol. The baboon model, which uses non-human primates that are genetically or evolutionarily close to humans and have a long life span, may be more informative when considering the full spectrum of liver damage in humans.

The mechanism by which alcohol induces disorders of the heart muscle (cardiomyopathies) has also been studied in animal models. This research has shown that alcohol acutely decreases the synthesis of contractile proteins in the heart and has suggested mechanisms for this decrease. Such studies have also explained the mechanisms by which chronic alcohol exposure damages heart muscle (Patel et al. 1997). Recently, a chicken model of alcohol-induced cardiomyopathy has been developed, which appears to mimic the human condition more closely, providing a new tool to study the mechanism of alcohol’s effects (Morris et al. 1999). These models add significantly to human epidemiological studies, because researchers can assess the mechanisms of alcohol-induced heart damage while controlling confounding factors and the amount of alcohol administered or ingested.

Human studies have found low to moderate alcohol intake to have a beneficial effect on the heart. Animal models have been developed recently to study the mechanism of this effect (e.g., Miyamae et al. 1998).

### Alcohol-Related Behavior

Researchers have also attempted to create animal models of various human alcohol-related behaviors. For example, many studies have investigated alcohol-associated aggression in rats and mice (e.g., Fish et al. 1999; Miczek et al. 1997), providing evidence of the physiological and neurochemical changes associated with increased aggression. These findings led to the development of therapies, such as selective serotonin reuptake inhibitors (compounds that prolong the activity of the neurotransmitter serotonin), which may be effective in reducing the incidence of alcohol-associated violence. However, the drugs used for management of violence have only a nominal effect in reducing human alcohol intake (Hoffman and Tabakoff 1999).

## Animal Models of Alcohol Intake

The studies described above represent attempts to generate animal models displaying face validity for alcohol-induced pathological changes in humans. Animal models of alcohol intake have also been developed, including a number of animal models of excessive alcohol intake (Weiss and Koob 1991). These models of alcohol-seeking behavior attempt to demonstrate the reinforcing (pleasurable) properties of alcohol, which are thought to play a key role in human alcohol use. Many of these models are embraced because they appear to have face validity, but this may be misleading; after all, it is difficult to identify the impetus for a behavior in a rodent or a nonhuman primate and to fully represent the human condition. These models may, however, have predictive validity, and may also be valuable for determining the neurochemical and molecular pathways that contribute to alcohol use.

One method of assessing alcohol intake uses animals that are given a choice between alcohol and another fluid. For example, in the two-bottle choice method, rats are allowed an unrestricted choice between alcohol and water 24 hours per day. Alternatively, access to alcohol, either alone or with a choice of another fluid, such as water, can be restricted to a certain period of time during the day. Several approaches have been used to produce a reliable demonstration of voluntary alcohol drinking. These include alcohol acclimatization (i.e., providing gradually increasing alcohol concentrations), taste adulteration (i.e., addition of sweeteners to the alcohol solution), and the use of prandial models, which take advantage of the postmeal drinking seen in rats.

In all of these models, animals are allowed to consume alcohol voluntarily. If nothing else, these models have shown that heterogeneous populations of animals, like humans, display a large range of alcohol consumption and that alcohol is consumed more avidly if it is contained in sweetened solutions (i.e., these models have face validity). These studies have also shown that genetic manipulation by inbreeding or selective breeding can produce animals displaying very defined (either high or low) alcohol preference.

An inherent limitation of the two-bottle choice method and others of this type is that it is difficult to use them to demonstrate the animal’s motivation to obtain alcohol. Motivation is demonstrated, however, in operant models (i.e., models in which the animal must perform a certain task to receive alcohol) of alcohol intake. In these models, an animal is trained to press a lever and receive alcohol, generally by the oral route. By adjusting the number of lever presses needed to receive the alcohol “reward,” one can assess how hard an animal will “work” to receive the alcohol. In some instances, the alcohol is administered via injection directly into the stomach through surgically implanted tubes (i.e., intragastric self-administration). This method has been used to avoid the influence of taste (i.e., to assure that alcohol is being administered by the animal for its pharmacological properties). The alcohol may also be self-administered directly to the brain. Using this procedure, researchers can identify the brain regions involved in alcohol’s reinforcing effects and minimize confounding factors such as metabolism (Meisch and Lemaire 1993).

One of the most successful operant models for inducing relatively high levels of oral alcohol intake by rodents uses a sweet solution (e.g., saccharin) to introduce animals to alcohol, after which the concentration of sweetener is gradually reduced. This procedure produces reliable operant responding for alcohol within a reasonable length of time (i.e., weeks) and can generate blood alcohol levels high enough to affect the animal’s behavior. A modification of this model has been described in which food- and water-sated rats that had been operantly trained to administer alcohol orally were allowed to obtain water or alcohol by responding on one of two levers. This paradigm addresses several key issues regarding alcohol reinforcement: (1) alcohol intake is maintained by pharmacological motivation, rather than factors related to appetite or thirst, and (2) alcohol changes and maintains the lever-pressing behavior, which functions to provide alcohol to the animal. The maintenance of lever-pressing behavior is interpreted as an indication that alcohol is functioning as a reinforcer (Weiss and Koob 1991). Using this model, certain rats have been shown to display a significant preference for alcohol over water and to achieve high blood alcohol concentrations.

Operant alcohol self-administration has not only been used to assess the reinforcing effect of alcohol, but also to model the craving for alcohol experienced by abstinent alcoholics. When animals have been drinking alcohol regularly and are then subjected to a period of forced abstinence from alcohol, they show a reliable increase in alcohol intake when alcohol is again made available (i.e., the alcohol deprivation effect). Whether this apparently enhanced motivation to ingest alcohol is an accurate model of craving in humans is not clear. However, this model does have significant predictive validity; the drugs now used to reduce craving and relapse in humans (e.g., acamprosate and naltrexone) can also block the increased responding associated with the alcohol deprivation effect in animal models.

Another method used to assess the reinforcing properties of alcohol is conditioned place preference. For this procedure, the animal receives alcohol and is then placed in a distinctive environment. The animal associates the effect of alcohol with that environment. If the effect of alcohol is pleasant (reinforcing) to the animal, it will later choose the distinctive environment over another environment when given a choice. In contrast, an animal that finds the effect of alcohol aversive will spend less time in the alcohol-associated environment. Mice have been found to demonstrate a conditioned place preference for alcohol in a number of studies, although an alcohol place preference is more difficult to demonstrate in rats and seems to require preexposure of the rats to alcohol. Rats are more likely to show aversion to alcohol in this model. The reason for this difference is not known, but again illustrates the importance of the choice of animal models for mimicking aspects of human behavior.

Drug discrimination procedures provide another method for ascertaining alcohol’s pharmacological properties and sites of action. In the simplest application of the procedure, animals are trained to respond for a food reward using a particular lever when alcohol (i.e., the reference drug) is administered and another lever when water is administered. The animal is then given an agent (i.e., a test drug) that is known to act at a specific receptor (i.e., a binding site for a specific brain chemical) and is allowed to respond on either lever. If the animal perceives that the effect of the test drug is similar to that of alcohol, the animal will respond with the alcohol-associated lever. Although the receptor sites identified with this model may or may not play a role in mediating the reinforcing effect of alcohol, this model can be used to identify targets for the development of therapies to interfere with various actions of alcohol.

Most or all of the animal models outlined above probably do not have face validity, but many have substantial predictive validity. Animal models with a great degree of face validity are primarily nonhuman primates, living in a social situation, that have access to alcohol. These animals are expensive to maintain and require substantial expertise on the part of the investigator, but the paradigm has the potential to provide relevant behavioral, genetic, pharmacological, and neurochemical information. For example, personality characteristics and the influence of rearing experiences on alcohol consumption have been studied in rhesus monkeys living in social groups, and these monkeys could also be evaluated for neurochemical characteristics, such as the activity of neurotransmitter systems in the brain (Higley and Linnoila 1997). The results of studies using these models appear to provide important parallels to the human situation and should have considerable predictive validity as well as face validity.

## Selective Breeding and Other Genetic Models

It is generally accepted that there are both environmental and genetic influences, and interactions between these factors, on the development of human alcohol dependence (i.e., alcoholism). The strong evidence for a role of genetics in human alcoholism (e.g., twin and adoption studies; alcoholism risk in individuals with family histories; subtypes of alcoholism) has led to a substantial effort to identify “alcoholism-related” genes by using genetic animal models.

For this type of research, it is desirable for investigators to use animals whose genetic make-up (i.e., genome) has been well characterized, as well as animals for which a known relationship exists between the organization of the animal genome and the human genome. The mouse best meets these criteria, currently, but the genomic information on other animal species (e.g., rat or monkey) is being rapidly accumulated. When the genomic information on the other species is available, it will contribute to the enhanced growth of genetically focused animal models. Mouse models of various alcohol-related behaviors have been successfully used to identify portions of the mouse genome associated with these behaviors and, thus, to indicate analogous regions of the human genome that may be associated with the same behaviors (e.g., alcohol’s effects on coordination). Although mice are generally used as models for alcohol research, a recent study found that the organization of the human genome is closer to that of the chicken than to that of the mouse (Burt et al. 1999). This finding raises the question of whether scientists should be modeling alcohol-related behaviors in chickens rather than mice. Differences in behavior between avians and mammals, however, may outweigh this possible advantage. For example, chickens would not be good models for studying infants’ genetic sensitivity to brain damage from alcohol ingested through mother’s milk.

The first question that arises when searching for genetic determinants for alcoholism-related behaviors is, which alcohol-related behaviors are the most relevant? The behaviors that have been most widely studied include sensitivity to alcohol’s effects; changes that occur in response to chronic alcohol exposure, such as tolerance, physical dependence, and sensitization; and alcohol “preference,” or intake.

Selective breeding produced some of the earliest genetic models of alcohol-related behaviors and is still generating important information (Crabbe and Belknap 1992; McBride and Li 1998). In this technique, mice or rats are bred to create lines of animals that are sensitive or insensitive to a particular effect of alcohol. Most of the selectively bred lines currently available originate from genetically heterogeneous foundation populations whose individuals were screened for sensitivity to alcohol’s effects. Breeding pairs are chosen for extreme sensitivity or insensitivity, and produce offspring that are also screened and selectively bred. This process is continued for several generations, until highly sensitive and insensitive lines are produced. Theoretically, if inbreeding is avoided or minimized, the genes that influence the selected trait will be “fixed” while genes unrelated to the selected trait will be randomly distributed in the selected lines. Therefore, if the selected lines differ in a biochemical or behavioral trait other than the one for which they have been selected (i.e., a “correlated trait”), it can be concluded that a common set of genes influences the two traits.

Selective breeding has been used extensively to study alcohol-related behaviors, and lines have been bred that differ in sensitivity to the hypnotic effect of alcohol (e.g., long-sleep and short-sleep mice); the hypothermic effect of alcohol (e.g., COLD and HOT mice); the locomotor stimulatory effect of alcohol (e.g., FAST and SLOW mice); alcohol withdrawal seizures (e.g., withdrawal seizure-prone [WSP] and resistant [WSR] mice); and acute functional alcohol tolerance (a measure of tolerance to alcohol that occurs within one testing session as opposed to tolerance development that occurs over several days of alcohol treatment and testing) (e.g., HAFT and LAFT mice). In addition, at least five lines of rats that differ in their alcohol intake (preference) have been bred. In all cases, animals have been bred bidirectionally (i.e., pairs of selected lines were generated that displayed high and low responses to the particular effect of alcohol being studied, or high and low preference for alcohol), and in most cases replicate lines have been bred to allow rapid verification of any differences between one pair of selected lines. Selected lines have provided a great deal of information regarding the genetic and biochemical basis of alcohol-related responses. In particular, rat lines selected for alcohol preference have been shown to ingest alcohol for its pharmacological properties, to differ in correlated traits such as sensitivity and tolerance to various alcohol effects, and to provide good predictive validity for identifying therapies to reduce alcohol intake (McBride and Li 1998).

There are a number of caveats associated with the selective breeding approach. For example, some inbreeding is unavoidable because of the relatively small population size in selective breeding studies, resulting in the fixation of genes unrelated to the selected trait. The estimation of genetic correlations, based on correlated responses to selection, is subject to a number of statistical as well as genetic considerations (Crabbe et al. 1990). It is also necessary to consider whether the behavior being selected (e.g., sensitivity to alcohol-induced loss of ability of an animal to right itself when placed on its back [“righting reflex”]) reflects primarily the effect of alcohol on the central nervous system, or whether the behavior may also be influenced by other factors, such as the mouse’s overall coordination, body weight, or body fat. A corollary of this issue is whether the behavior (e.g., the anesthetic effect of alcohol) that is measured in the selected species is relevant to human motivation for ingesting alcohol.

Other genetic models of alcohol-related behaviors include inbred strains, recombinant inbred strains, and transgenic/knock-out mice (Gora-Maslak et al. 1991; Wehner and Bowers 1995). An inbred strain is usually derived from successive brother-sister matings for at least 20 generations, resulting in a genetically identical group of animals. Individual differences among inbred mice are theoretically due exclusively to environmental factors, and when such factors are held constant, differences among inbred strains demonstrate genetic influence. The difficulty of holding environmental factors constant, however, was recently demonstrated in a study conducted by several different laboratories, in which a concerted effort was made to standardize the environment of the animals. Although the laboratories used animals obtained from the same sources and used the same testing procedures, they obtained differing results. The researchers concluded that only a strong genetic influence over a trait would negate the environmental influences (Crabbe et al. 1999*a*). That is, if the mice are genetically identical, the differences are due to unidentified environmental variables.

Nevertheless, inbred strains, particularly strains of mice, have been successfully used to reveal genetic influences on responses to alcohol and to many other drugs. The advantages of using inbred strains include the ability to compare data collected in different laboratories (in spite of the environmental factors), the stability of strain responses over time, and the ability to compare acute and chronic responses to alcohol or other drugs in different groups of mice. Disadvantages include the fact that it is difficult to generalize results from any one inbred strain to the mouse population as a whole (much less the human population) and that it is necessary to use a large number of inbred strains in order to generate valid genetic correlations. However, many groups have made substantial progress in investigating the genetics of alcohol-related behaviors using inbred strains.

Recombinant inbred (RI) strains are derived from a pair of standard inbred strains. Within an inbred strain, each individual has two copies of the same form (i.e., allele) of each gene. Any two inbred strains differ at some percentage of the chromosome, randomly distributed across the complete set of chromosomes. The two progenitor strains are interbred to generate a genetically identical F_1_ population; at all areas of the chromosome where the progenitors differed, offspring receive one allele from each parent. Next, the F_1_s are randomly bred to produce the genetically heterogeneous F_2_ population. In this population, parts of the parental chromosomes have recombined. Inbred strains are generated from the F_2_ population by randomly chosen brother-sister mating for at least 20 generations. This inbreeding produces animals that are genetically identical for one or the other progenitor’s alleles at all locations on the chromosome. This process yields a unique pattern of recombinations of the parental chromosomes in each RI strain (Crabbe and Belknap 1992).

The original use of this animal model was to provide a powerful tool for establishing genetic associations and identifying major gene effects. For example, if upon testing a group of RI strains for a response to alcohol, a researcher finds that the response of each RI strain resembles one of the responses of a progenitor, this suggests that a single gene is exerting an important influence. Once this major gene effect has been identified, linkage can be determined by matching the allele pattern for the locus (i.e., location on a chromosome) of the identified gene across the RI strains (the “strain distribution pattern”) with the strain distribution pattern for previously mapped genetic markers. If the strain distribution patterns for the new locus and a mapped marker are the same, the new locus must be closely linked to the marker.

In addition to major gene effects, however, RI strains can also be used to identify and locate genes that have smaller influences on the measured trait. This is an important advance, because most phenotypic characteristics (i.e., traits influenced by genetic factors), such as behavioral responses to alcohol, are not “all-or-none,” but vary over a continuous range of values. These quantitative traits are generally influenced by multiple genes (i.e., polygenic), and each gene may have only a small influence on the phenotype. In order to identify the gene loci associated with quantitative traits (i.e., quantitative trait loci [QTL]), the allelic variation is correlated with the phenotypic variability in the RI strains. In other words, the behavioral or biochemical alcohol-related responses from the strains can be correlated with genetic markers, each scored as 0 or 1 to represent alleles from the two progenitor strains. Significant correlations indicate associations between the marker(s) and the quantitative responses (i.e., a QTL). Furthermore, overlapping QTLs for two traits indicate genetic correlations between the traits. This approach depends on the localization of a large number of markers on the genome and provides the starting point for identifying particular (candidate) genes within an identified QTL region. In animals, a relatively large number of QTLs for alcohol-related behaviors have been identified (Crabbe et al. 1999*b*). The leap from QTL identification to gene identification will depend on the methodology for refining the position of the QTL (Rikke and Johnson 1998), which is currently being developed.

The caveats associated with QTL analysis are essentially statistical, (e.g., the occurrence of false positives and false negatives). The level of power chosen to detect the statistical significance of the observed correlations allows researchers to strike a balance between type I (i.e., false positive) and type II (i.e., false negative) errors. In general, QTLs detected using RI strains are considered “provisional” and need to be confirmed. Confirmation may be accomplished, for example, by analysis of an F_2_ population, which can provide a larger number of animals, and therefore greater statistical strength, or by the use of congenic animals (i.e., inbred animals that carry a small segment of a chromosome from another strain).

As markers on the human genome are mapped, QTL analysis can also be carried out in humans. Recently, researchers have reported several “susceptibility loci for alcohol dependence” in humans (Reich et al. 1998). Their identification is subject to the caveats noted above. The complex phenotype of alcoholism in humans and the polygenic influences on this disorder suggest that genetic animal models will continue to provide crucial information for application to the human analysis. A current challenge is to create a categorization of behavioral-physiological correlates of alcoholism in humans for which animal models can provide both face and predictive validity. Such categorization will provide for more relevant comparisons of genetic data obtained from humans and other animals.

Other strategies for evaluating a gene’s contribution to alcohol-related behaviors include the production of genetically engineered transgenic and knock-out animals. These strategies, unlike QTL analysis, are not aimed at finding new genes, but at evaluating the importance of candidate genes (genes believed to contribute to the development of a particular disease). Candidate genes are generally identified through analyses of the neurochemical or biochemical determinants of alcohol-related responses. To create a transgenic mouse, a foreign gene is integrated into the mouse’s own genetic material. Transgenic animals overexpress the foreign gene, and the influence of the gene on their responses to alcohol can be determined. This is a powerful technique for assessing the influence of a candidate gene, although there are a number of caveats associated with it. For example, until recently there has been no control over where in the chromosome the foreign gene, or transgene, is integrated. One way to circumvent this problem is to create a number of transgenic lines. Recently developed techniques allow researchers to target the integration of the transgene. Errors of interpretation can also occur if tissue-specific promoters are not used and the transgene is expressed in all tissues. As more tissue-specific promoters are being identified, the alteration of gene expression in specific cells and tissues will be facilitated. With respect to alcohol-related behaviors and alcoholism, the influence of any single gene manipulation must be of a great enough magnitude that it can be reliably assessed. The issue of genetic background is also important, both for transgenic animals and the knock-out mice described below, because the magnitude of effects may vary depending on the animal’s own genetic makeup. The creation of transgenic animals using several different backgrounds may provide some control for this problem.

Knock-out mice are mice in which a gene has been inactivated or altered. Studies with knock-out mice are subject to many of the problems mentioned for the transgenic mice, some of which are currently being overcome as described. In addition, because some genes are essential for development, animals in which these critical genes are deleted do not survive. In other cases, the gene mutation may prompt compensatory adaptations during development that may overcome the effect of the alteration. These compensatory changes, rather than the gene of interest, can therefore influence the alcohol-related behaviors. Techniques to alleviate these problems include methods for conditional excision of the genes (i.e., conditional knock-outs), which could be programmed to occur in the adult animal. Models are also being developed in which more subtle mutations, which control the level of gene expression, can be introduced. Several knock-out mice have been used in alcohol studies to date, including protein kinase C (PKC) and dopamine and serotonin (D_2_ and 5–HT_1b_) receptor knock-outs, in which sensitivity to alcohol and alcohol drinking behavior have been assessed. Although these studies have revealed some interesting differences between knockout mice and wild-type (normal) mice, the differences have not always been replicable, perhaps reflecting the importance of compensatory adaptations during development as well as gene targeting and genetic background.

## Dose, Chronology, and Route of Administration Issues

In all animal models of alcohol-related behaviors, investigators need to consider the alcohol doses used, the duration and spacing of alcohol administration, and the route of alcohol administration, in light of the influence of these parameters on the data obtained and its relevance to the human situation. For example, because the metabolic rate of alcohol varies among species, it is necessary to assure that the doses of alcohol used will generate blood and brain alcohol levels that can produce pharmacological effects. This has been a particular issue in studies using oral alcohol self-administration, where the amount of alcohol taken in may not exceed the metabolic capacity of the animal and no significant accumulation of alcohol in brain or other tissues may occur. In addition, different species have different sensitivities to the effects of alcohol. For example, mice appear to be less sensitive than rats to alcohol effects, such that similar doses (and similar brain and blood alcohol levels) may produce different behavioral responses in the two species. This can be an important consideration when applying the results from animal model studies to humans. The use of end-points, such as the development of alcohol tolerance or physical dependence in animals, can provide a rationale for choosing a particular dose or duration of alcohol exposure, even if the mode or duration of alcohol administration does not resemble that seen for humans.

The route of alcohol administration must also be considered. Feeding animals alcohol in a liquid diet as the sole source of nutrition differs from the normal pattern of alcohol intake in humans, even though alcohol is taken orally. However, this method can be used reliably to generate tolerance, dependence, and the alcohol withdrawal effect. Similarly, models using continuous gastric infusion of ethanol are very different from the method of alcohol intake by humans. However, by controlling for nutritional status and allowing high levels of alcohol intake, such models can be useful for understanding how alcohol ingestion can lead to organ damage. Operant techniques for oral self-administration of alcohol may or may not resemble the manner of alcohol intake by humans, and alcohol administration by injection or implanted tube is certainly different from the means of intake by humans. Even voluntary alcohol intake in a two-bottle choice situation is quite different from human patterns of alcohol intake. However, these models may provide insight into the neurophysiological basis for excessive alcohol intake.

## Conclusion

Thus, as already stated by McClearn (1988), the experimental paradigms generated by researchers working with humans or animals are significantly constrained by the characteristics of the subjects, including the competence of a particular species to perform certain tasks. Equipment and conceptual constraints may limit generalizability. The refinement of animal models needs to continue until the hypotheses arising from animal models can be ethically and practically tested on humans.
